# Pseudorabies virus infection increases the permeability of the mammalian respiratory barrier to facilitate *Pasteurella multocida* infection

**DOI:** 10.1128/msphere.00297-24

**Published:** 2024-07-23

**Authors:** Dajun Zhang, Lin Lin, Jie Yang, Qingjie Lv, Mixue Wang, Lin Hua, Keshan Zhang, Huanchun Chen, Bin Wu, Zhong Peng

**Affiliations:** 1 National Key Laboratory of Agricultural Microbiology, College of Veterinary Medicine, Huazhong Agricultural University, Wuhan, China; 2 Hubei Hongshan Laboratory, Wuhan, China; 3 Frontiers Science Center for Animal Breeding and Sustainable Production, The Cooperative Innovation Center for Sustainable Pig Production, Wuhan, China; 4 Department of Veterinary Medicine, College of Life Science and Engineering, Foshan University, Foshan, China; University of Michigan, Ann Arbor, Michigan, USA

**Keywords:** pseudorabies virus infection, *Pasteurella multocida *infection, interactions, epithelial permeability, respiratory epithelial cells, mice

## Abstract

Interaction between viruses and bacteria during the development of infectious diseases is a complex question that requires continuous study. In this study, we explored the interactions between pseudorabies virus (PRV) and *Pasteurella multocida* (PM), which are recognized as the primary and secondary agents of porcine respiratory disease complex (PRDC), respectively. *In vivo* tests using mouse models demonstrated that intranasal inoculation with PRV at a sublethal dose induced disruption of murine respiratory barrier and promoted the invasion and damages caused by PM through respiratory infection. Inoculation with PRV also disrupted the barrier function of murine and porcine respiratory epithelial cells, and accelerated the adherence and invasion of PM to the cells. In mechanism, PRV infection resulted in decreased expression of tight junction proteins (ZO-1, occludin) and adherens junction proteins (β-catenin, E-cadherin) between neighboring respiratory epithelial cells. Additionally, PRV inoculation at an early stage downregulated multiple biological processes contributing to epithelial adhesion and barrier functions while upregulating signals beneficial for respiratory barrier disruption (e.g., the HIF-1α signaling). Furthermore, PRV infection also stimulated the upregulation of cellular receptors (CAM5, ICAM2, ACAN, and DSCAM) that promote bacterial adherence. The data presented in this study provide insights into the understanding of virus-bacteria interactions in PRDC and may also contribute to understanding the mechanisms of secondary infections caused by different respiratory viruses (e.g., influenza virus and SARS-CoV-2) in both medical and veterinary medicine.

**IMPORTANCE:**

Co-infections caused by viral and bacterial agents are common in both medical and veterinary medicine, but the related mechanisms are not fully understood. This study investigated the interactions between the zoonotic pathogens PRV and PM during the development of respiratory infections in both cell and mouse models, and reported the possible mechanisms which included: (i) the primary infection of PRV may induce the disruption and/or damage of mammal respiratory barrier, thereby contributing to the invasion of PM; (ii) PRV infection at early stage accelerates the transcription and/or expression of several cellular receptors that are beneficial for bacterial adherence. This study may shed a light on understanding the mechanisms on the secondary infection of PM promoted by different respiratory viruses (e.g., influenza virus and SARS-CoV-2) in both medical and veterinary medicine.

## INTRODUCTION

Respiratory infections are a leading cause of disease and economic burden in both humans and animals (1, 2). Herpesvirus 1, a double-stranded linear DNA virus, and *Pasteurella multocida* (PM), a gram-negative bacterial pathogen, are capable of causing respiratory infections in a wide range of host species, including humans (3, 4). Notably, the co-detection of these two agents is common in pigs (5). Both suid herpesvirus type 1 [also known as pseudorabies virus (PRV)] and PM can independently cause respiratory infections in pigs (6, 7). They also recognized as important primary and secondary pathogen, respectively, in porcine respiratory disease complex (PRDC) (8). The incidence of PRDC is attributed to the interactions among viral, bacterial, and adverse environmental and management conditions, with the combination of primary and opportunistic infectious agents being recognized as the most significant factor (8). However, the interactions between these primary and secondary opportunistic agents remain unclear.

The respiratory barrier serves as the initial defense against respiratory pathogens (9). The integrity of the respiratory barrier relies on the tight junctions (e.g., ZO-1 and occludin) and adherens junctions (e.g., β-catenin and E-cadherin) between adjacent epithelial cells (10). Disrupting the respiratory barrier is a crucial strategy employed by respiratory pathogens to invade hosts and cause infections (11, 12). Previous studies conducted in mouse models and respiratory epithelial cell models during PM infection have supported this notion (11, 13). However, it remains to be investigated whether primary agents (generally are the viruses) contribute to the invasion of PM during the development of PRDC and respiratory diseases in other animal species, considering that PM is commonly regarded as a secondary opportunistic agent in these diseases (8).

In this study, we utilized mice and mammalian respiratory epithelial cells as models to investigate the interactions between PRV and PM. Our findings demonstrated that respiratory infection with a sublethal dose of PRV induced the disruption of the mammalian respiratory barrier, thereby facilitating PM infection. This study provides valuable insights into understanding the interactions between primary and secondary opportunistic agents not only in PRDC but also in the development of respiratory diseases associated with the co-infection of viruses and bacteria in both humans and animals.

## MATERIALS AND METHODS

### Bacteria, virus, and cell culture

PM HN05 (GenBank accession no. PPVF00000000) was isolated from the trachea of a pig that died from respiratory diseases. PRV SMX is a clinical isolate collected by our laboratory from pigs during the 2012 outbreak, and its lethal dose (LD) to mice was 19.5TCID_50_ per mouse (14). Unless specified otherwise, PM strains were cultured on tryptic soy agar (TSA) or in tryptic soy broth (Becton, Dickinson and Company, Sparks, MD, US) supplemented with 5% newborn calf serum. PRV was propagated in PK15 cells (ATCC CCL33) maintained in Dulbecco’s modified Eagle’s medium (DMEM) supplemented with 10% fetal bovine serum (FBS; Gibco, US). NPTr cells (gifted by Prof. Hongbo Zhou at Huazhong Agricultural University) were maintained in DMEM supplemented with 10% FBS under a 5% CO_2_ atmosphere. MLE-12 cells were maintained in a DMEM-Ham’s F-12 medium nutrient mixture (Gibco, US) containing 2% FBS, 1% GlutaMAX-1, 1 M 1% HEPES buffer solution, 1% penicillin-streptomycin, 1% insulin-transferrin-selenium, 10 nM hydrocortisone, and 10 nM estradiol at 37°C under a 5% CO_2_ atmosphere.

### Mouse experiments

**Fig 1:**
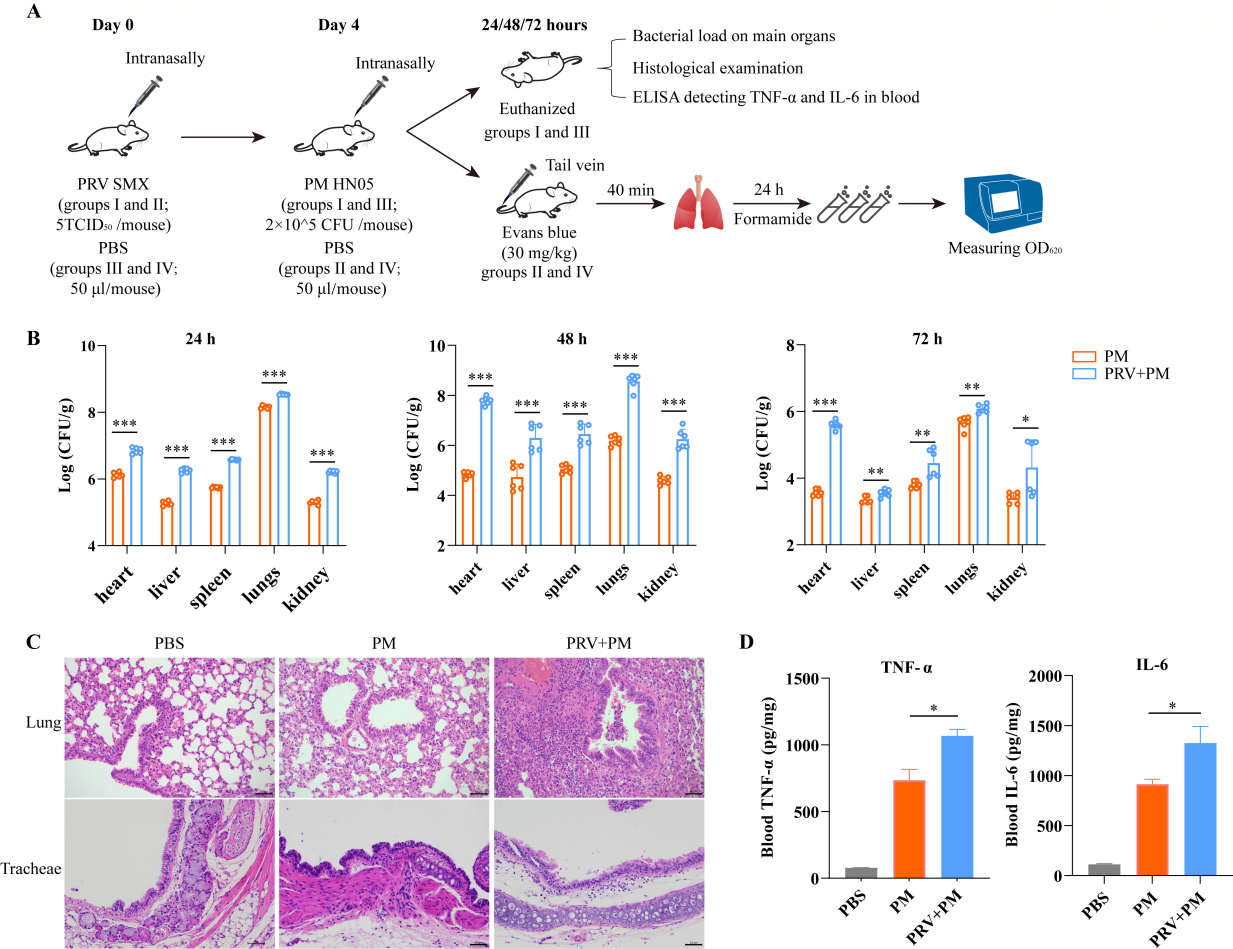
Mouse experiments to assess the effect of PRV inoculation at a sublethal dose on PM infection post intranasal inoculation. (**A**) Study design of the mouse experiments. Experimental mice were intranasally inoculated with PRV at a sublethal dose (5TCID_50_ per mouse) or PBS, followed by intranasal inoculation with PM (2 × 10^5^ CFU per mouse). To assess the effect of PRV infection on the permeability of the murine respiratory barrier, experimental mice intranasally inoculated with PRV at a sublethal dose or PBS received an injection of Evans blue (EB; 30 mg/kg) through the tail vein, followed by the extraction of EB in murine tracheae and lungs using formamide. (**B**) The numbers of PM strains recovered from different organs (hearts, livers, spleens, lungs, and kidneys) of mice inoculated with PRV or PBS. The data were presented as mean ± SD. Statistical significance was indicated as *P* < 0.05 (*), *P* < 0.01 (**), or *P* < 0.001 (***). (**C**) Histological examinations of lungs and tracheae collected from healthy mice, PBS-treated mice challenged with PM, and/or PRV-treated mice challenged with PM. Thickened and congestive alveolar walls were observed in the lungs of PBS-treated mice challenged with PM compared to the lungs of the healthy mice. However, more severe damages were observed in the lungs of PRV-treated mice challenged with PM, including withered and collapsed alveoli, exfoliated and necrotic epithelial cells, and infiltration of a large amount of inflammatory cells (bars = 50 µm). The structure of tracheae in PBS-treated mice challenged with PM was abnormal compared to the tracheae of healthy mice, with infiltration of inflammatory cells. However, the mucosal layer of the tracheae in PRV-treated mice challenged with PM was significantly thinner, with more exfoliated and necrotic mucosal epithelial cells, and loose edema and obvious inflammatory cell infiltration were observed in the submucosa of the tissue (bars = 50 µm). (**D**) The titers of TNF-α and IL-6 in the bloods collected from healthy mice, PBS-treated mice challenged with PM, and/or PRV-treated mice challenged with PM. The data were presented as mean ± SD. Statistical significance was indicated as *P* < 0.05 (*).

The mouse experiment was conducted at the Laboratory Animal Center of Huazhong Agricultural University (Wuhan, China). Briefly, 64 Kunming mice that were 8 weeks old were randomly divided into four groups (groups I–IV), each group comprising either 19 (groups I and III) or 13 mice (groups II and IV). Each mouse in groups I and II was intranasally inoculated with a sublethal dose of PRV SMX (5TCID_50_/mouse; 0.25LD), while those in groups III and IV received an intranasal administration of PBS (50 µL per mouse) following the same protocol. At 4 days post inoculation (dpi), each mouse in groups I and III was intranasally inoculated with PM HN05 at a dose of 2 × 10^5^ CFU (Fig. 1A). Mice in the remaining two groups were intranasally inoculated with PBS. Morbidity and mortality were recorded twice daily after PM inoculation. At 24, 48, and 72 h post PM inoculation (hpi), three mice randomly selected from groups I and III were euthanized, and their hearts, livers, spleens, lungs, and kidneys were collected for PM recovery and counting. Lungs and tracheae were also used for histological examinations. The production of TNF-α and IL-6 in murine lungs and blood was determined using real-time quantitative PCR (qPCR) and enzyme-linked immunosorbent assay (ELISA), respectively.

To assess the effect of PRV infection at a sublethal dose on murine respiratory barrier, three randomly selected mice from groups II and IV received a systemic injection of Evans blue (EB; 30 mg/kg; Sigma, St. Louis, USA) via the tail vein at 4 days post PRV or PBS inoculation (Fig. 1A). After 40 min, the mice were euthanized, and EB in mouse tracheae and lungs was extracted in 1 mL formamide at overnight 55°C, as previously described (11). The supernatants were harvested after centrifugation at 12,000 rpm for 6 min, and the absorbance of optical density (OD) values at 620 nm (OD_620_) was measured. At the same time point (48 h post PRV or PBS inoculation), murine tracheae were collected for histological examinations and/or transmission electron microscope checking.

### Bacterial adherence and invasion assays

To investigate the potential contribution of PRV to PM infection, bacterial adherence and invasion assays were conducted using MLE-12 and/or NPTr models. Prior to the experiments, the susceptibility of MLE-12 and/or NPTr was evaluated. Monolayers of MLE-12 or NPTr cells (>80% confluence) were inoculated with PRV SMX at different multiplicities of infection (MOIs) (0.1, 0.5, 1). The cells were then incubated at 37°C under a 5% CO_2_ atmosphere to observe the occurrence of cytopathic effects at various time points. Based on these evaluations, an MOI of 0.1 was chosen for subsequent experiments. Following this, PRV SMX was inoculated into the cell monolayers and incubated for 2 h. Control cells were treated with PBS. Afterward, unabsorbed free virus was removed by washing using PBS. The PRV-/PBS-treated cells were subsequently exposed to PM HN05 at an MOI of 100. The cells were incubated 37°C for 6 h under a 5% CO_2_ atmosphere. Unattached bacteria were removed by washing using PBS, and the cells were lysed for 10 min at 37°C, using 0.1% Triton-X (Wako, Mie, Japan) in PBS. Finally, the cell lysates were subjected to a series of 10-fold dilutions, and the dilutions were plated on TSA agars containing 5% newborn calf serum to determine bacterial counts.

In addition to the adherence assay, after removing the unattached bacteria, the cells were treated with ampicillin and kanamycin (100 ng/µL) for 30 min at 4°C to eliminate extracellular bacteria. The invading bacteria were then counted by plating the 10-fold diluted cell lysates on TSA agars containing 5% newborn calf serum. All above experiments were repeated three times separately.

### Dextran-based *trans*-well permeability assays

Dextran-based *trans*-well permeability assays were performed following previously described methods (11). Briefly, approximately 1 × 10^5^ cells (MLE-12 or NPTr) were seeded onto cell culture inserts (Labselect, Hefei, China) placed in the wells of a 24-well plate (Corning, USA). The cells were cultured in antibiotic-free medium (as described in “Bacteria, virus, and cell culture,” above) for 36 h to establish dense monolayers. Subsequently, 200 µL of antibiotic-free medium containing PRV SMX (MOI = 0.1) and 70 kDa fluorescein isothiocyanate (FITC; Sigma) at a concentration of 10 µM were added to the wells. The plate was then incubated at 37°C for 12 h under a 5% CO_2_ atmosphere. Following this, 100 µL of medium from the basal chamber was transferred to a black-well plate (Greiner Bio-One, Germany) for fluorescence measurement using a Victor Nivo multimode plate reader (PerkinElmer, Waltham, USA) at an excitation/emission wavelength of 490/520 nm. Dextran permeability was determined based on the results, as described previously (15).

### Fluorescence assay

The fluorescence assay was performed following a previously described protocol (16). Briefly, PM HN05 (10^9^ CFU/mL) was suspended in 0.1 M sodium bicarbonate buffer (pH = 9.0). The bacteria were then incubated with 2 µg/mL FITC (Solarbio, Beijing, China) saturated in dimethyl sulfoxide for 60 min at room temperature. After incubation, the FITC-labeled bacteria were washed using PBS and adjusted to an MOI of 100 in antibiotic-free DMEM. Subsequently, NPTr or MLE-12 cells, with or without PRV inoculation (MOI = 0.1) for 2 h were exposed to the FITC-labeled PM in the DMEM medium. The cells were examined under an inverted fluorescence microscope (Nikon, Japan).

### RNA-Seq and bioinformatic analysis

A monolayer of NPTr cells (2 × 10^5^ cells per well) on a six-well plate (Corning, USA) was incubated with PRV (MOI = 0.1) for 2 h at 37°C. After removing the unabsorbed free virus, the cells were further incubated at 37°C for another 12 h. PBS-treated cells were used as controls. Total RNAs were extracted from the PRV-infected cells and control cells using the Trizol reagent protocol (Invitrogen, Waltham, USA). The extracted RNA samples were assessed for purity, concentration, integrity, and quantity using a NanoDrop spectrophotometer (Thermo Scientific, USA), Qubit 4.0 (Thermo Scientific, USA), in combination with the Agilent 2100/4200 system (Agilent Technologies, USA). Afterward, 3 μg of RNA was used as input material, and sequencing libraries were prepared using a NEBNext UltraTM RNA Library Prep Kit for Illumina (NEB, Ipswich, USA) following the manufacturer’s recommendations. Finally, libraries were sequenced on DNBSEQ-T7 platform using the PE150 model at Bioyi Biotechnology Co., Ltd. (Wuhan, China). The gene expression analysis in PRV-infected cells and control cells was performed using DESeq2 (17). The resulting *P*-values were adjusted using the Benjamini and Hochberg approach (18) to control the false discovery rate. Genes with an adjust *P*-value (*P*adj) ≤0.05 and the expression of an absolute fold change (|FoldChange|) ≥2 between the two samples (PRV-infected cells vs control cells) were considered as differentially expressed, following the previously described criteria (13).

### qPCR

To validate the RNA-Seq data, NPTr cells were treated with PRV SMX, and total RNAs were extracted and assessed as described in “RNA-Seq and bioinformatic analysis,” above. The extracted RNAs were then used to synthesize cDNAs using a PrimeScript RT Master Mix Kit (TaKaRa, Kusatsu, Japan). The synthesized cDNA served as the template for performing qPCR assays. Primers using to detect the transcription level of selected genes in different groups of cells are provided in Table S1. The relative transcription levels of genes were calculated as a ratio of the target gene to the reference gene using the formula 2^−(ΔΔCt)^ (13). The transcription level of several selected genes in NPTr cells with or without PRV infection was determined using qPCR assays with primers listed in Table S1. In MLE-12 cell models, MLE-12 cells were infected by PRV as described in “RNA-Seq and bioinformatic analysis,” above, and total RNAs from PRV-infected cells and the control cells (without PRV infection) were extracted to synthesize cDNAs using the commercial PrimeScript RT Master Mix Kit (Vazyme, Nanjing, China). Transcription levels of several selected genes in different groups of cells were also detected by qPCR assays with primers listed in Table S1.

### Western blot analysis

NPTr and/or MLE-12 cells were inoculated with PRV SMX as described above. Cells with or without PRV infection were lysed in RIPA buffer (Beyotime, Shanghai, China) containing protease inhibitor cocktail (Sigma-Aldrich, Burlington, USA). The lysates were then subjected to centrifugation at 4°C, 12,000 rpm for 10 min to remove insoluble debris. The protein concentration in the lysates was determined using a commercial enhanced BCA protein assay kit (Beyotime, Shanghai, China). The cell lysates were separated on 10% sodium dodecyl sulfate polyacrylamide gel electrophoresis and transferred onto polyvinylidene difluoride membranes (Bio-Rad, Hercules, USA). The blots were blocked in 5% BSA in Tris-buffered saline with Tween 20 for 1 h at room temperature. Subsequently, the blots were incubated with ZO-1 polyclonal antibody (catalog no. ab216880, Abcam, Cambridge, USA), β-catenin polyclonal antibody (catalog no. 21773-1-AP, Proteintech, Rosemont, USA), or GAPDH monoclonal antibody (1:20,000) (catalog no. 60004-1-lg, Proteintech, USA) at 4°C overnight. After washing, the blots were incubated with species-specific horseradish peroxidase-conjugated antibodies and finally visualized with enhanced chemiluminescence reagents (Beyotime, Shanghai, China). All western blots (WB) were quantified using ImageJ software (version 5.2.1) (Bio-Rad, Hercules, USA), and the results were analyzed as the relative immunoreactivity of each protein normalized to the respective loading control.

### Statistical analysis

Statistical analysis was performed through the “multiple *t*-tests” strategy in GraphPad Prism8.0 (GraphPad Software, San Diego, CA) to compare the means of multiple groups or conditions to assess differences between them. Data represent mean ± SD. The significance level was set at *P* < 0.05 (*), *P* < 0.01 (**), or *P* < 0.001 (***).

## RESULTS

### Intranasal inoculation of PRV at a sublethal dose promoted colonization and damage caused by PM in mice following upper respiratory tract infection

Compared to the PBS-treated mice, the PRV-treated mice exhibited a significantly higher (*P* < 0.05) number of PM strains recovered from the hearts, livers, spleens, lungs, and kidneys (Fig. 1B). Histological examinations also revealed more severe damages on the lungs and tracheae of PRV-treated mice compared to the PBS-treated mice (Fig. 1C). In addition, inoculation with PM led to the production of a higher level of cytokines (TNF-α and IL-6) in murine blood in PRV-treated mice than that in PBS-treated mice (Fig. 1D). The above findings indicated that intranasal inoculation of PRV at a sublethal dose promotes the colonization and damages caused by PM in mice after upper respiratory tract infection.

### Intranasal inoculation of PRV at a sublethal dose induced the damage of murine respiratory barrier

**Fig 2:**
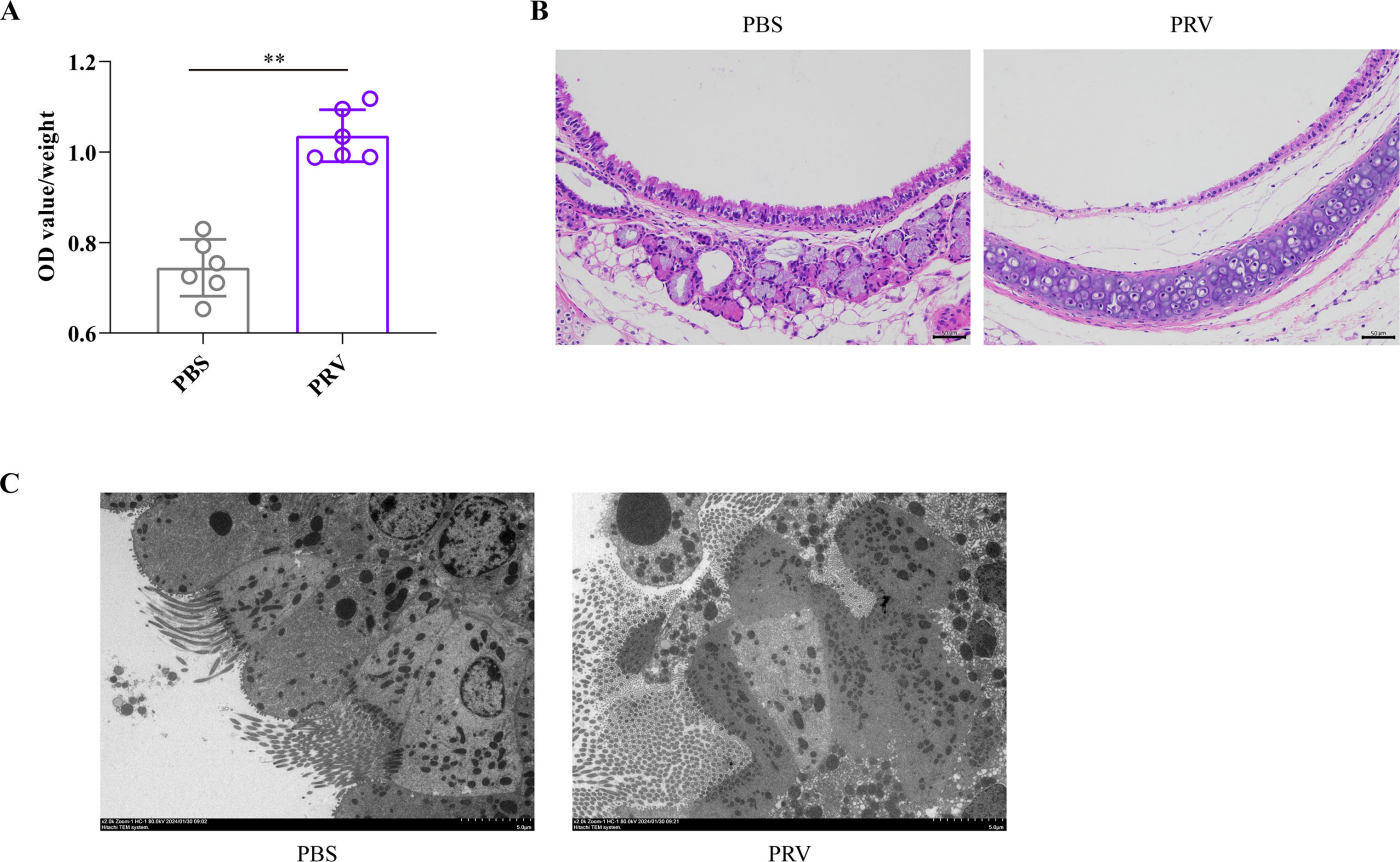
Mouse experiments to assess the effect of PRV inoculation at a sublethal dose on the damage of murine respiratory barrier. (**A**) Quantification of EB in tracheae and lungs collected from mice inoculated with PRV and PBS. The data were presented as mean ± SD. Statistical significance was indicated as *P* < 0.01 (**). (**B**) Histological examinations of the tracheae collected from healthy mice and PRV-inoculated mice. The structure of the bronchus was abnormal in the tracheae collected from PRV-inoculated mice compared to those from the healthy mice. The mucosal layer of the tissue became thinner, and the epithelial cells of the mucosa were shed and necrotic. In addition, loose edema and a small amount of inflammatory cells were seen in the submucosa (bars = 50 µm). (**C**) Transmission electron microscope examination of the tracheae collected from healthy mice and PRV-inoculated mice. The tracheal villi of PRV-inoculated mice were broken, and the tight connections were not clear (bars = 5 µm).

To investigate whether respiratory inoculation of PRV at a sublethal dose affects the integrity of the murine respiratory barrier, we assessed the permeability of the respiratory barrier using EB dye (Fig. 1A). The results revealed that PRV-treated mice had higher levels of EB in their lungs and tracheae compared to the PBS-treated mice (Fig. 2A). This indicates that the integrity of the respiratory barrier is indeed increased in PRV-treated mice. To further confirm this finding, we collected tracheae from both PRV-treated and PBS-treated mice for histological examination and transmission electron microscope observation. The results demonstrated that intranasal inoculation of PRV at a sublethal dose led to damage in the barrier function of the murine trachea (Fig. 2B and C).

### PRV inoculation promoted PM adherence and invasion to respiratory epithelial cells

**Fig 3:**
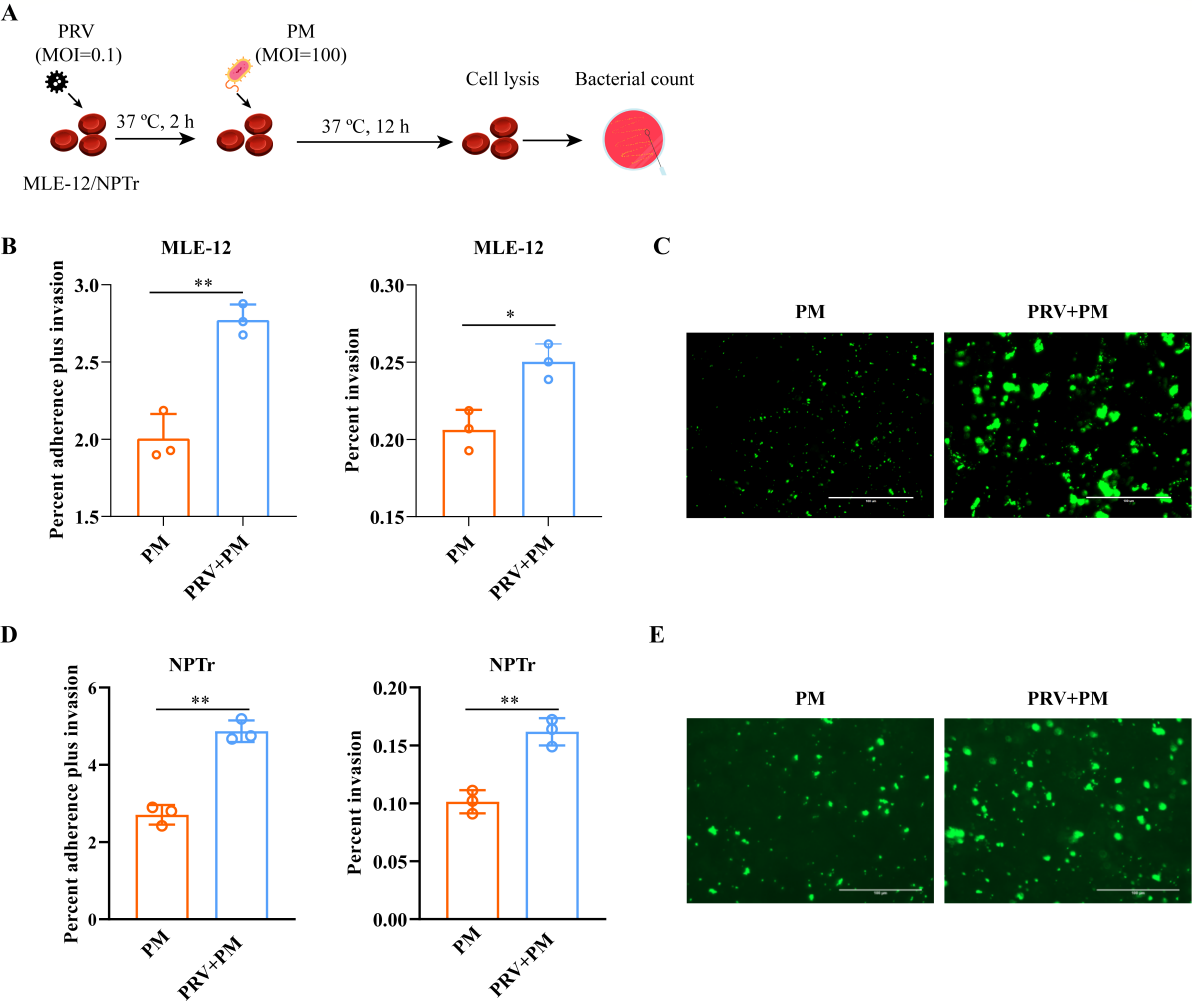
PRV enhances PM adherence and invasion of murine respiratory epithelial cells (MLE-12) and porcine respiratory epithelial cells (NPTr). (**A**) Study design. PM strains were inoculated 2 h after PRV inoculation. The cells were then incubated at 37°C for 12 h. Finally, the cells were lysed for bacterial counting. (**B**) Column charts showing the percentage of PM adherence and invasion in MLE-12 cells pre-inoculated with or without PRV. The data were presented as mean ± SD. Statistical significance was indicated as *P* < 0.05 (*) or *P* < 0.01 (**). (**C**) Fluorescence microscope images of FITC-labeled PM (green) adhering to MLE-12 cells pre-inoculated with or without PRV (bars = 100 µm). (**D**) Column charts showing the percentage of PM adherence and invasion in NPTr cells pre-inoculated with or without PRV. The data were presented as mean ± SD. Statistical significance was indicated as *P* < 0.01 (**). (**E**) Fluorescence microscope images of FITC-labeled PM (green) adhering to NPTr cells pre-inoculated with or without PRV (bars = 100 µm).

To gain further insights into the mechanisms underlying the interaction between PRV and PM, *in vitro* models utilizing respiratory epithelial cells from mice (MLE-12) and pigs (NPTr) were employed (Fig. 3A). Bacterial adherence and invasion assays were conducted, and the results revealed that a higher number of PM strains adhered to and/or invaded the PRV-treated cells compared to the PBS-treated cells (Fig. 3B through E). These findings indicate that PRV inoculation promotes the adherence and invasion of PM to respiratory epithelial cells.

### PRV inoculation disrupted the respiratory epithelial barrier by downregulating tight and adherens junctions

**Fig 4:**
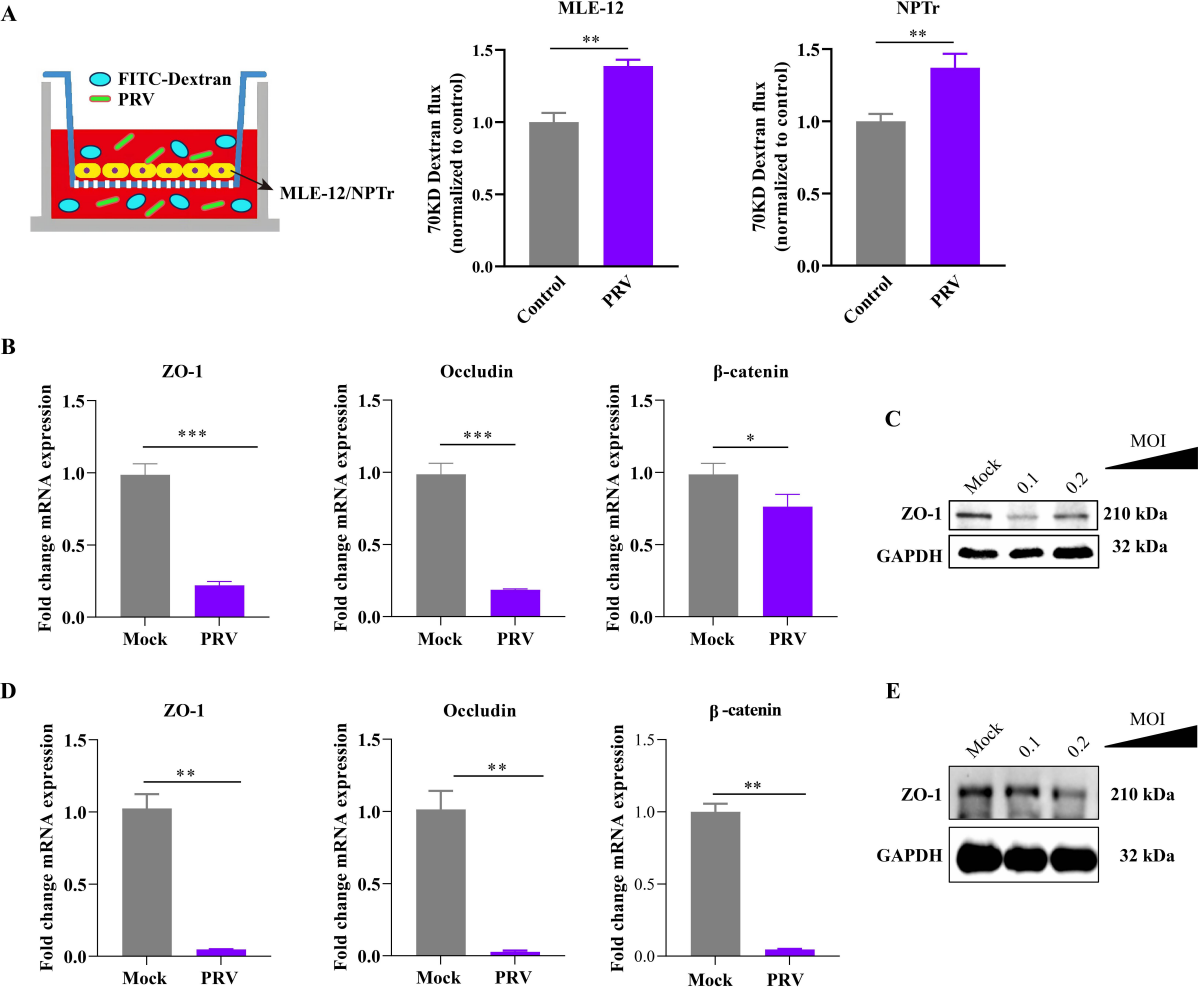
*In vitro* tests demonstrate the influence of PRV infection at an early stage on the barrier function formed by different mammal respiratory epithelial cells. (**A**) Dextran-based *trans*-well permeability assay showing the increase in vascular permeability of murine respiratory epithelial cells (MLE-12) or porcine respiratory epithelial cells (NPTr) induced by PRV infection at an early stage. Cells inoculated with PBS were included as control for the assay. (**B**) Column charts showing the transcription levels of tight junction proteins (ZO-1, occludin) and adherens junction proteins (β-catenin) between neighboring cells quantified by qPCR in PRV-inoculated MLE-12 cells compared to the mock cells. The data were presented as mean ± SD. Statistical significance was indicated as *P* < 0.05 (*) or *P* < 0.001 (***). (**C**) The expression of ZO-1 between neighboring cells quantified by western blot in PRV-inoculated MLE-12 cells compared to the mock cells. (**D**) Column charts showing the transcription levels of tight junction proteins (ZO-1, occludin) and adherens junction proteins (β-catenin) between neighboring cells quantified by qPCR in PRV-inoculated NPTr cells compared to the mock cells. The data were presented as mean ± SD. Statistical significance was indicated as *P* < 0.01 (**). (**E**) The expression of ZO-1 between neighboring cells quantified by western blot in PRV-inoculated NPTr cells compared to mock cells.

To further investigate the influence of PRV inoculation on the barrier function of respiratory epithelial cells, dextran-based *trans*-well permeability assays were conducted (Fig. 4A). The results demonstrated that inoculation of PRV at an MOI of 0.1 decreased the integrity of the barrier function formed by both MLE-12 and NPTr cells (Fig. 4A). To understand the underlying mechanisms, the expression levels of important tight junction proteins (ZO-1, occludin) and adherens junction protein (β-catenin) were assessed using qPCR and/or western blot analysis. The results indicated a decreased level of these molecules in PRV-treated cells compared to the PBS-treated cells (Fig. 4B through E). These findings suggest that PRV infection disrupts the respiratory epithelial barrier by downregulating tight junctions and adherens junctions.

### PRV infection regulated the expression of many molecules in respiratory epithelial cells contributing to bacterial adherence and invasion, and the maintenance of barrier function

**Fig 5:**
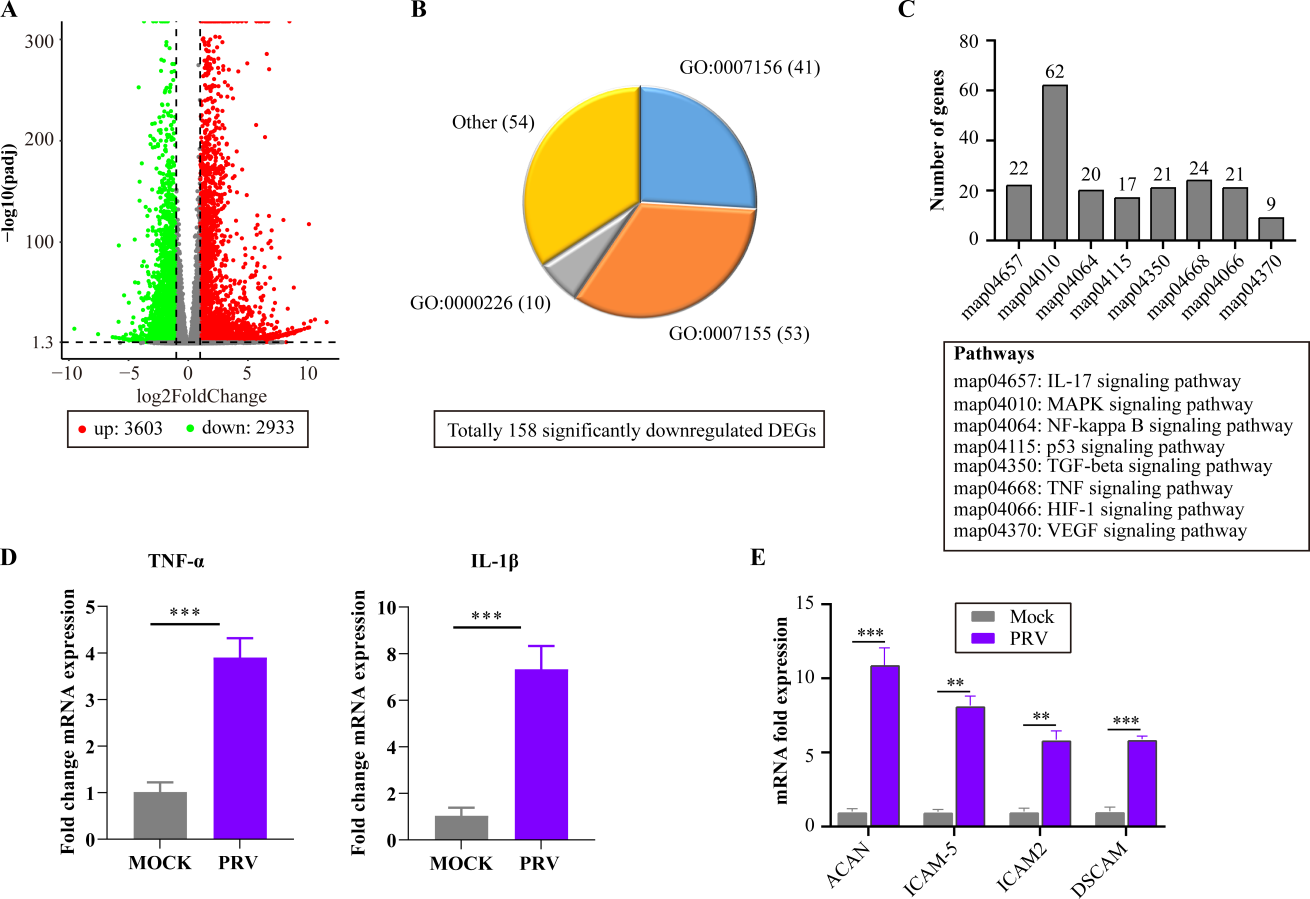
Changes in gene expression in mammalian respiratory epithelial cells induced by PRV infection. (**A**) Dot plots showing the number of differentially expressed genes in PRV-infected pig respiratory epithelial cells (NPTr) compared to mock cells. (**B**) Pie charts showing the number of significantly downregulated DEGs in NPTr cells induced by PRV infection that enriched in different Gene Ontology functional catalogs. (**C**) Column charts showing the number of upregulated DEGs in NPTr cells induced by PRV infection that enriched in KEGG pathways related to the increase of the permeability of respiratory epithelial cells. (**D**) Column charts showing the transcription levels of TNF-α and IL-1β quantified by qPCR in PRV-infected NPTr cells compared to mock cells. The data were presented as mean ± SD. Statistical significance was indicated as *P* < 0.01 (***). (**E**) Column charts showing the transcription levels of cellular receptors beneficial for bacterial adherence in NPTr cells quantified by qPCR in PRV-infected cells compared to mock cells. The data were presented as mean ± SD. Statistical significance was indicated as *P* < 0.01 (**) or *P* < 0.001 (***).

To further explore the mechanisms of PRV infection on the barrier function of respiratory epithelial cells and its contribution to bacterial infection, RNA-Seq was conducted using RNAs extracted from NPTr monolayers inoculated with PRV (MOI = 0.1) or PBS. A total of 6,536 differentially expressed genes (DEGs) were identified in PRV-treated cells compared to PBS-treated cells, including 3,603 upregulated ones and 2,933 downregulated ones (|FoldChange| ≥2, *P*adj ≤0.05) (Fig. 5A). The significantly downregulated DEGs (*n* = 158) were primarily enriched in Gene Ontology (GO) biological processes associated with epithelial adhesion and barrier functions, including cell adhesion (GO:0007155), microtubule cytoskeleton organization (GO:0000226), and homophilic cell adhesion via plasma membrane adhesion molecules (GO:0007156) (Fig. 5B; Table S2). Furthermore, genes involved in various signaling pathways contributing to the disruption of tissue barriers, such as the HIF-1/VEGFA signaling pathway (map04066/map04370), were also upregulated in PRV-treated cells (Fig. 5C; Table S3). The above findings suggest that early-stage PRV infection increases the epithelial permeability of respiratory barrier.

Both transcriptome sequencing and qPCR validation showed upregulation of genes encoding inflammatory factors (TNF-α and IL-1β) in PRV-treated cells compared to PBS-treated cells (Fig. 5D). Consistently, DEGs enriched in pathways related to inflammatory response, such as the TNF signaling pathway (map04668), TGF-β signaling pathway (map04350), MAPK signaling pathway (map04010), and IL-17 signaling pathway (map04657), were also upregulated in PRV-treated cells compared to PBS-treated cells (Fig. 5C; Table S3). Additionally, the transcription of molecules beneficial for bacterial adherence, including ACAN, ICAM5, ICAM2, and DSCAM, was upregulated in PRV-treated cells compared to PBS-treated cells (Fig. 5E). These findings suggest that early-stage PRV infection may induce inflammatory reactions in respiratory epithelial cells and increase the expression of molecules that facilitate bacterial adherence, thereby contributing to damage to the respiratory barrier and bacterial invasion.

## DISCUSSION

The interaction between different agents, especially viruses and bacteria, in the development of infectious diseases is a complex topic that requires continuous study in both human and veterinary medicine (19). This is particularly true in farm animals, such as pigs and cattle, where mixed infections or coinfections are more common (20, 21). In the pig industry, PRDC is a typical disease that involves mixed infections caused by different pathogens (8). In this study, we investigated the interaction between a known primary pathogen, PRV, and a known secondary pathogen, PM, in mouse and cell models. While mixed infections of PRV and PM are more common in pigs, we applied mouse as a substitute *in vivo* model due to the complexity of the internal environments of pigs and the challenges in maintaining optimal experimental condition. Additionally, rodents are the natural hosts of PRV and PM (22, 23), and mice are commonly used as model animal in laboratory research (11, 24).

Using the mouse model, we demonstrated that intranasal inoculation of PRV at a sublethal dose resulted in an increase in PM invasion and the permeability of the respiratory barrier in mice (Fig. 1 and 2). The respiratory epithelial barrier, being one of the most important tissue barriers in mammals, serves as the first line of defense against invading pathogens in the respiratory tract (25, 26). A previous study has demonstrated that the integrity of the respiratory epithelium is crucial in the host’s innate defense against primary alphaherpesvirus infections using equine herpesvirus type 1 as a model (27). In this study, we observed PRV infection disrupted the integrity of the respiratory epithelial barrier, consequently facilitating the invasion of PM following intranasal inoculation. It is noteworthy that PM is a part of the normal flora in the upper respiratory tract of pigs (3), and respiratory exposure is a common route of PRV infection on pig farms (28). Therefore, secondary infection of PM may occur in pigs after PRV proliferation, leading to damages in swine respiratory tracts. Although it is yet to be determined whether PRV disrupts the integrity of the swine respiratory epithelial barrier at the early stage of infection, our previous study involving pig challenge demonstrated clear damages to the swine respiratory tracts caused by PRV (29). Therefore, a possible mechanism by which PRV promotes PM infection could be through the disruption and/or damage of the swine respiratory barrier induced by PRV, thereby facilitating PM invasion.

The increase in the permeability of the respiratory barrier during the early stage of PRV infection was demonstrated in both murine and porcine respiratory epithelial cell models using dextran-based *trans*-well permeability assays (Fig. 1A, 3, and 4), which are commonly used to evaluate changes in barrier function in different epithelial cells (11, 15). Tight junctions (e.g., ZO-1 and occludin) and adherens junctions (e.g., β-catenin and E-cadherin) between neighboring epithelial cells are known to play a critical role in maintaining the integrity of tissue barriers (30–32). In this study, we detected the transcription and/or expression of these molecules using qPCR, WB, and/or RNS-Seq, and observed a decrease in their levels in different respiratory epithelial cells (MLE-12, NPTr) after early-stage exposure to PRV (Fig. 5). These further confirm that PRV infection during the early stage has the ability to disrupt the respiratory epithelial barrier. Consistently, our RNA-Seq results also revealed downregulation of multiple biological processes involved in epithelial adhesion and barrier functions, while signals associated with respiratory barrier disruption [e.g., the HIF-1α signaling (11, 15, 33)] were upregulated in PRV-treated cells compared to the control cells. Additionally, RNA-Seq showed that PRV inoculation during the early stage of infection led to an increase in the transcription/expression of several cellular receptors [e.g., ICAM5, ICAM2, ACAN, and DSCAM (Fig. 5E)] that are known to facilitate bacterial adherence (34, 35). This could also explain why PRV infection promotes the adherence of PM to respiratory cells (Fig. 3).

**Fig 6:**
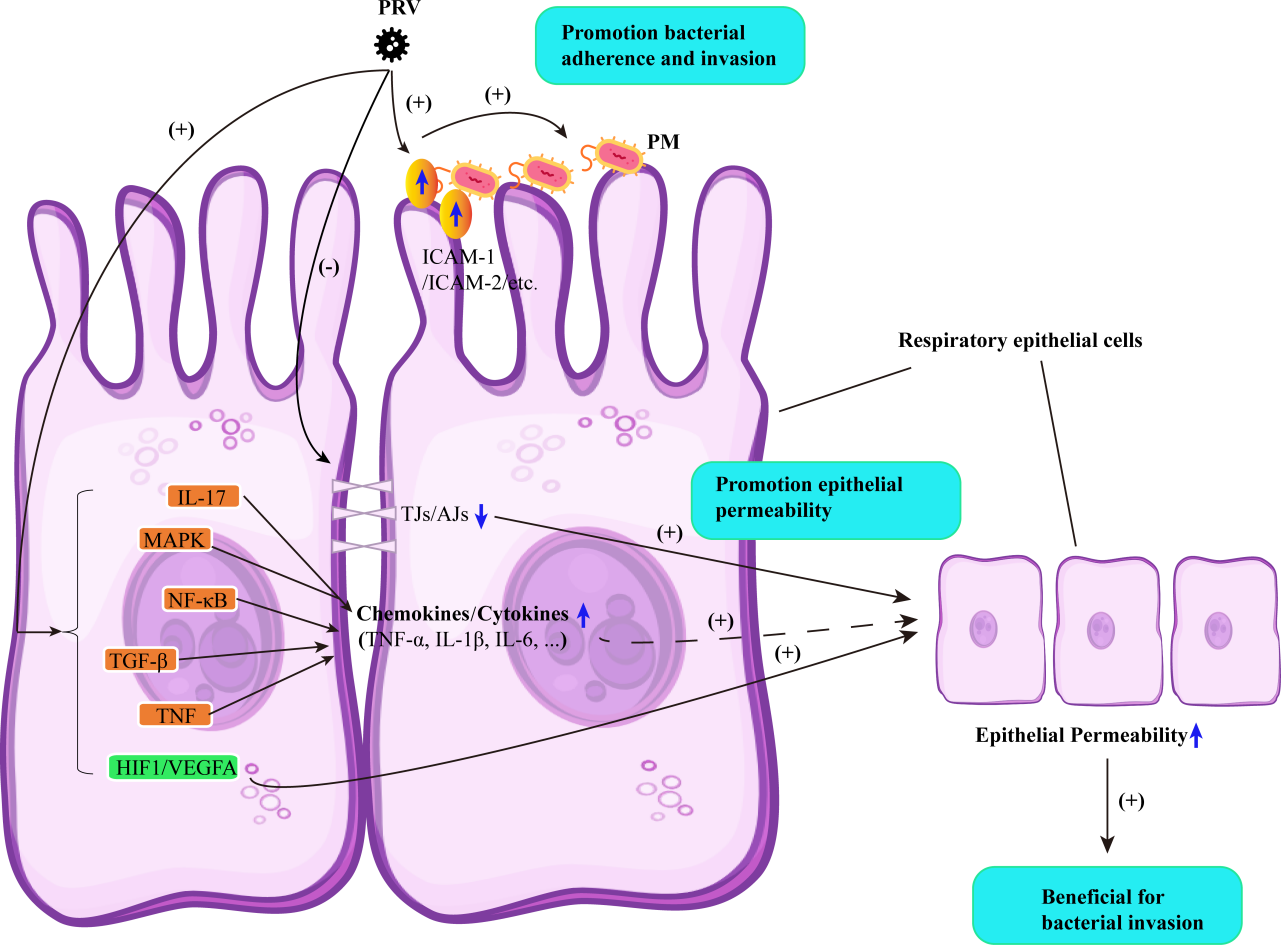
Proposed mechanisms of PRV infection at an early stage promoting PM invasion of the mammal respiratory barrier. PRV infection induces an increase in the permeability of respiratory epithelial cells by stimulating the decrease of tight junctions and adherens junctions, thereby facilitating PM invasion. Several signals induced by PRV infection, particularly the pro-inflammatory signals and the HIF-1/VEGFA, may also participate in this process. Additionally, PRV infection promotes the transcription and/or expression of several molecules on the cells, such as ICAM5, ICAM2, ACAN, and DSCAM, which enhance PM adherence.

In conclusion, we reported for the first time a potential interaction mechanism between PRV and PM in PRDC. Although we acknowledge the interactions between these two agents during the development of PRDC are more complex and require further studies to explore additional details, our data presented in this study partially revealed a mechanism by which PRV infection promotes secondary PM infection. First, the primary PRV infection induces disruption and/or damage to the porcine respiratory barrier, thereby facilitating PM invasion. Second, early-stage PRV infection accelerates the transcription and/or expression of several cellular receptors, such as ICAM5, ICAM2, ACAN, and DSCAM, which enhances PM adherence (Fig. 6). The findings of this study provide insights into virus-bacteria interactions in PRDC and shed light on the mechanisms underlying secondary PM infection promoted by different respiratory viruses [e.g., influenza virus (36) and SARS-CoV-2 (37)] in both medical and veterinary medicine.

## Data Availability

Data from the transcriptome sequencing have been deposited into the NCBI Sequence Read Archive (SRA) database under the BioProject PRJNA871929; the accession numbers are SRR22159395, SRR22159393, SRR22159391, SRR22159389, SRR22159387, and SRR22159385. Remaining data that support the findings are openly available in Figshare at 10.6084/m9.figshare.25391155.

## SUPPLEMENTAL MATERIAL

Table S1 msphere.00297-24-s0001.docx Primers used for qPCR assays in this study.

Table S2 msphere.00297-24-s0002.xlsx GO functions of significantly downregulated DEGs in PRV-infected NPTr cells compared to the control cells.

Table S3 msphere.00297-24-s0003.xlsx KEGG functions of significantly upregulated DEGs in PRV-infected NPTr cells compared to the control cells.

ASM does not own the copyrights to Supplemental Material that may be linked to, or accessed through, an article. The authors have granted ASM a non-exclusive, world-wide license to publish the Supplemental Material files. Please contact the corresponding author directly for reuse.
