# Supplementary material for: Pseudorabies virus infection increases the permeability of the mammalian respiratory barrier to facilitate Pasteurella multocida infection
Source: mSphere. 2024 Jul 23;9(8):e00297-24. doi: 10.1128/msphere.00297-24 (PMC11351098; doi:10.1128/msphere.00297-24)
Supplement: Table S1 — Primers used for qPCR assays in this study. [file msphere.00297-24-s0001.docx]

**Additional file 1.** Primers used for qPCR assays in this study.

| **Primers** | **Sequences (5’-3’)** | **Target genes** |
| --- | --- | --- |
| Pig-ICAM5-F | GTGGGCGAGAACTTTACCCT | Porcine ICAM5 |
| Pig-ICAM5-R | AAATTGGCCCCATGGTCCTC |  |
| Pig-ICAM2-F | ATGGTTCACCATCGAGTGCC | Porcine ICAM2 |
| Pig-ICAM2-R | TGTGGTGCCCATCTTTCCTG |  |
| Pig-ACAN-F | AAGGTTGCTACGGGGACAAG | Porcine ACAN |
| Pig-ACAN-R | GACCTCACCCTCCATCTCCT |  |
| Pig-DSCAM-F | GTGGGCGAGAACTTTACCCT | Porcine DSCAM |
| Pig-DSCAM-R | ATCCCGTGTGTTGTGGTCTC |  |
| Pig-TJP3-F | CACCCAACCAGGTCTCTCTCA | Porcine TJP3 |
| Pig-TJP3-R | CTCTGTCCTCCCCACCTGTTT |  |
| Pig-CLDN3-F | GACTACGTATGAGGGGGCAG | Porcine CLDN3 |
| Pig-CLDN3-R | CATCTGGGTGGACTGGTCTC |  |
| Pig-CLDN8-F | TCCATCCAAGGGCAGAATGG | Porcine CLDN8 |
| Pig-CLDN8-R | GTTGCTTCCAATGAAGGCGG |  |
| Pig-BCL9L-F | TGTCAAGGGCCTATGGAGAC | Porcine BCL9L |
| Pig-BCL9L-R | GGGAGAAGAATGGTGTGGGT |  |
| Pig-CDH1-F | GCACCAACCCTCCTGAGTGT | Porcine CDH1 |
| Pig-CDH1-R | AAAGTTTCCAATTTCATCAGGATTG |  |
| Pig-OCLN-F | ATCAACAAAGGCAACTCT | Porcine OCLN |
| Pig-OCLN-R | GCAGCAGCCATGTACTCT |  |
| Pig-TNF-F | CCAGACCAAGGTCAACCTCC | Porcine TNF-α |
| Pig-TNF-R | TCCCAGGTAGATGGGTTCGT |  |
| Pig-IL6-F | ACAAAGCCACCACCCCTAAC | Porcine IL-6 |
| Pig-IL6-R | CGTGGACGGCATCAATCTCA |  |
| Pig-ZO1-F | ACCCACCAAACCCACCAA | Porcine ZO-1 |
| Pig-ZO1-R | CCATCTCTTGCTGCCAAACTATC |  |
| Pig-E-cadherin-F | GCACCAACCCTCCTGAGTGT | Porcine E-cadherin |
| Pig-E-cadherin-R | AAAGTTTCCAATTTCATCAGGATTG |  |
| Pig-Occludin-F | ATCAACAAAGGCAACTCT | Porcine Occludin |
| Pig-Occludin-R | GCAGCAGCCATGTACTCT |  |
| Pig-β-catenin-F | GCCTTCACTACGGACTACC | Porcine β-catenin |
| Pig-β-catenin-R | ATCCTGATGAGCACGAACC |  |
| Pig-GAPDH-F | ACAGGGTGGTGGACCTCATG | Porcine GAPDH |
| Pig-GAPDH-R | GGGTCTGGGATGGAAACTGG |  |
| H-GAPDH-F | GAGTCAACGGATTTGGTCGT | Human GAPDH |
| H-GAPDH-R | GACAAGCTTCCCGTTCTCAG |  |
| H-IL-6-F | TTCTCCACAAGCGCCTTCGGTC | Human IL-6 |
| H-IL-6-R | TCTGTGTGGGGCGGCTACATCT |  |
| H-TNF-α-F | GTGCTTGTTCCTCAGCCTCT | Human TNF-α |
| H-TNF-α-R | CTACAGGCTTGTCACTCGGG |  |
| H-ZO-1-F | AAACCTGGGGCTGTCTCAAC | Human ZO-1 |
| H-ZO-1-R | ATTTCATGCTGGGCCGAAGA |  |
| H-β-catenin-F | GGAGGAAGGTCTGAGGAGCAG | Human β-catenin |
| H-β-catenin-R | TGTCCAACTCCATCAAATCAGCTTG |  |
| H-occludin-F | GGTCTAGGACGCAGCAGATT | Human occludin |
| H-occludin-R | GGCTGAGAGAGCATTGGTCG |  |
